# Differences in lower-extremity strength and power between elite hockey players with and without patellar asymptomatic tendon thickening; unilateral and bilateral comparative analysis

**DOI:** 10.3389/fspor.2026.1841375

**Published:** 2026-06-01

**Authors:** Sofia Ryman Augustsson, Ludvig Omdal Persson, Haris Pojskić

**Affiliations:** Department of Sport Science, Linnaeus University, Kalmar, Sweden

**Keywords:** athletes, ice hockey, isokinetic strength test, ultrasound imaging, Wattbike Peak 6 test

## Abstract

**Introduction:**

Existing research shows that while patellar tendinopathy in ice hockey players is linked to cumulative mechanical overload, structural tendon changes such as thickening or degeneration do not reliably correspond to pain or performance deficits, leaving the functional impact of asymptomatic tendon pathology unclear. Thus, the aim of this study was to identify differences in lower-extremity strength and power between elite ice hockey players with and without patellar asymptomatic tendon thickening using unilateral and bilateral comparisons.

**Methods:**

This cross-sectional study combined clinical screening, performance testing, and ultrasound imaging to characterize patellar tendon structure and lower-extremity function. Players were objectively classified into healthy (*n* = 8), unilateral (n = 8), or bilateral patellar asymptomatic tendon thickening (*n* = 12) groups based on patellar tendon thickness and width. Strength and power outcomes were assessed under controlled conditions and normalized to body mass to allow fair between-group comparisons. Isokinetic concentric strength was evaluated during a split squat performed on a robotic resistance device, while peak power output was measured using a six-second all-out cycling test on a calibrated ergometer.

**Results:**

Relative power output was significantly higher in the healthy group (20.3 ± 1.7 W·kg⁻¹) compared with the bilateral patellar asymptomatic tendon thickening group (18.6 ± 1.1 W·kg⁻¹; *p* = 0.047), whereas no between-group differences were observed for relative peak force or absolute power (all *p* > 0.45). No significant left-right differences in peak force were detected within any group (all *p* ≥ 0.12).

**Discussion:**

Asymptomatic bilateral patellar tendon thickening was associated with a modest but significant reduction in relative power output in elite hockey players, whereas unilateral tendon changes did not impair performance. These findings suggest that bilateral structural tendon alterations may negatively influence power-generating capacity even in the absence of clinical symptoms.

**Clinical Trial Registration:** https://clinicaltrials.gov/study/NCT07356596, PROSPERO NCT073566.

## 1 Introduction

Ice hockey is a high-intensity contact sport involving repeated short bouts of maximal effort, frequent collisions, and rapid substitutions (1). Optimal performance requires explosive power, efficient recovery, and resilience to external forces (2, 3). Key physical attributes include muscle strength, anaerobic and aerobic endurance, explosiveness, and joint mobility (1, 4). The lower- extremities (LE), particularly the hip and knee joints, are critical for force production (5) and are highly susceptible to injury (6–9). In sports requiring fast skating and rapid changes of direction, such as ice hockey, this vulnerability contributes to a high prevalence of overuse injuries, including patellar tendinopathy (10). Tendinopathy often develops from cumulative overload (11, 12), triggering tendon tissue changes such as collagen disorganization and reduced elasticity (13). The key pathophysiologic feature of tendinopathy is tendinosis, a degenerative condition marked by impaired tissue repair, progressive tendon degeneration, and absence of inflammatory cells, resulting from chronic repetitive strain without adequate recovery (14). Clinically, patellar tendinopathy presents as load-related anterior knee pain that can impair performance (15) and, in severe cases, lead to premature career termination (10, 16). Affected individuals demonstrate reduced knee-extensor, knee-flexor, and hip strength compared with asymptomatic controls (17). Imaging commonly reveals increased tendon thickness and neovascularization (18), with greater tendon thickness identified as a risk factor even in asymptomatic individuals (19, 20).

Although tendon thickening is characteristic of patellar tendinopathy, it may also reflect a normal adaptive response to mechanical loading (21, 22). For example, resistance training increase tendon cross-sectional area (CSA) and stiffness (21), highlighting the paradoxical role of mechanical loading as both a key driver of beneficial tendon adaptation and a contributor to tendon pathology and pain (22). Evidence suggests that increases in tendon stiffness are driven primarily by changes in tendon modulus, with additional contributions from CSA (21). Still, while sport performance impairments are clearly associated with pain, the relationship between pain and tendon pathology remains unclear (23). In patellar tendinopathy, pain does not consistently correlate with the extent of structural changes (20, 24) as athletes may exhibit marked tendon degeneration with minimal symptoms, while others experience substantial pain without pronounced pathology (24). This dissociation suggests that tendinosis may contribute to the development and persistence of patellar tendinopathy but is not the sole determinant of pain or functional limitation. Additionally, evidence linking pathological changes to physical performance is limited. One study reported poorer jump performance in individuals with thinner tendons and larger CSA, along with a negative association between CSA and knee-extension strength, possibly reflecting reduced quadriceps activation (25). Consequently, the impact of patellar tendon thickening on athletic performance remains unclear, particularly whether asymptomatic structural changes impair strength and power, capacities essential for rapid skating and acceleration. Therefore, this study aimed to identify differences in LE strength and power between elite hockey players with and without asymptomatic patellar tendon thickening (PTT) using unilateral and bilateral comparisons.

## 2 Methods

### 2.1 Study design and participants

**Figure 1:**
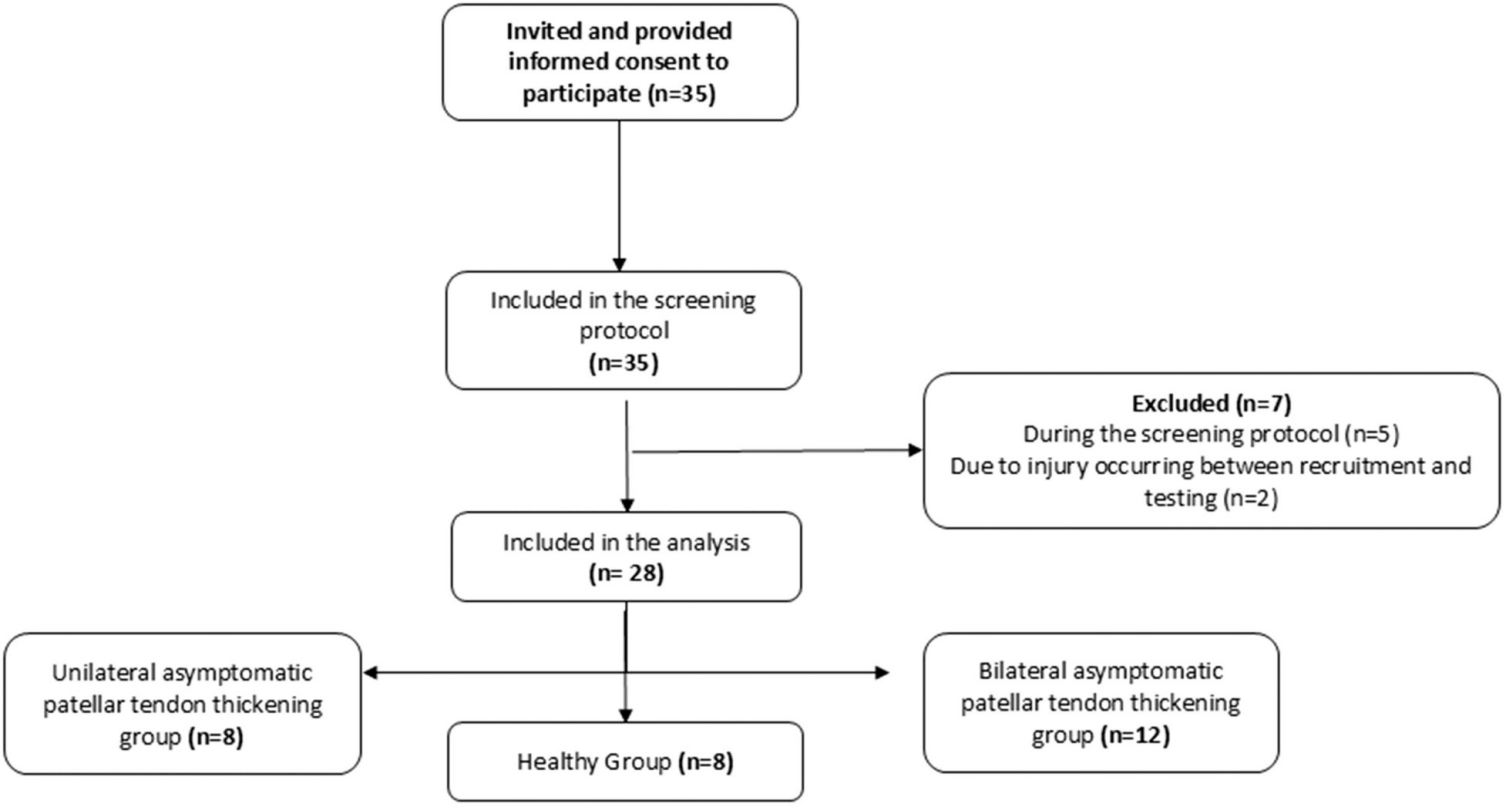
Flow chart of study participants.

This cross-sectional study, adhering to the STROBE statement (26), included 28 male players from the Swedish Hockey League (SHL) (Figure 1). A screening protocol was conducted by a licensed physiotherapist, who evaluated factors including instability, dysfunction, and pain. Only players without current or prior knee injuries that might affect LE function were included in the study. Based on ultrasound findings, players were divided into three groups: the healthy group (HG; *n* = 8), the unilateral PTT group (UPTTG; *n* = 8), and the bilateral PTT group (BPTTG; *n* = 12) (Table 1). The players trained 12 h per week, including four 2 h on-ice sessions and two 2 h off-ice sessions focused on strength, conditioning, and prehabilitation. Most weeks included two games. The study was performed in accordance with the declaration of Helsinki (27) and all players were informed of the study's purpose, potential benefits, and risks, and provided written consent prior to study start.

**Table 1:** Descriptive statistics (mean ± SD) and ANOVA differences between the healthy group and the groups with unilateral and bilateral PTT in age, anthropometrics, and patellar tendon thickness.

Variables	HG (*n* = 8)	UPTTG (*n* = 8)	BPTTG (*n* = 12)	*p*	*η*²
	Mean ± SD	Mean ± SD	Mean ± SD		
Age (years)	26.4 ± 5.9	24.9 **±** 4.5	24.6 **±** 3.8	0.611	0.039
Body Weight (kg)	86.4 ± 8.5	84.9 **±** 6.7	89.9 **±** 6.2	0.277	0.098
Body Height (cm)	183.8 ± 5.6	187.0 ± 5.3	185.3 ± 5.3	0.490	0.055
Body mass index (kg × m^−2^)	25.5 ± 1.2	24.3 ± 2.5	26.2 ± 1.9	0.124	0.154
LL-Patellar Tendon thickness (mm)	4.2 ± 0.4	4.6 **±** 0.7*	6.0 **±** 0.8*	<0.001	0.649
RL-Patellar Tendon thickness (mm)	3.9 ± 0.6	5.5 **±** 0.9*	5.8 **±** 0.8*	<0.001	0.542

HG, Healthy group; UPTTG, Unilateral patellar asymptomatic tendon thickening group; BPTTG, Bilateral patellar asymptomatic tendon thickening group; SD, standard deviation; RL, Right leg; LL, Left leg; p, one-way ANOVÁs probability of between-group differences with Bonferroni *post-hoc* pairwise comparison test; η², Eta-squared effect size of observed differences;.

* Significantly different from the healthy group at *p* < 0.05.

### 2.2 Experimental design and procedures

**Figure 2:**
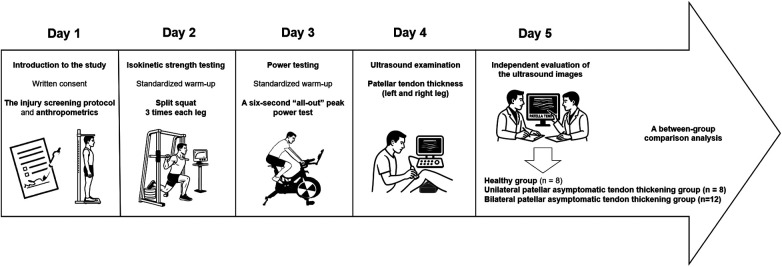
Experimental protocol.

To investigate whether asymptomatic structural changes impair hockey players' ability to generate strength and power, a between-group analysis based on patellar tendon thickness was conducted. The combination of ultrasound imaging with strength and cycling-based power assessments was chosen to capture both structural and functional aspects of tendon health. Tendon thickness was selected as the primary classification criterion because it represents a quantitative, objective, and reproducible parameter, with previous studies demonstrating higher reliability for quantitative ultrasound measures compared with qualitative assessments (28, 29). Because of testing time constraints originating from a compressed training schedule and to ensure consistent testing conditions for each player, this study was conducted over several consecutive days (Figure 2). On the first day, players were informed about the study, informed consent was obtained, and the injury screening and anthropometric measurements were done. On days two and three, strength and power testing were performed by one investigator. Given that patellar tendon thickness may depend on body mass index in athletes (30), strength and power tests were analyzed relative to their body mass. Players were asked to refrain from high-intensity training and to avoid sleep deprivation for at least 24 h before the testing sessions. On the fourth day, a second investigator, who was blinded to the test results, performed an ultrasound assessment of patellar tendon thickness in both legs. On the fifth day, two investigators independently analyzed ultrasound images to estimate tendon thickness in each leg. Based on the results and applying the cutoff value of patellar tendon thickness (>5 mm), the cohort was divided into three independent groups (i.e., healthy, unilateral, and bilateral asymptomatic tendon thickening groups). Lastly, a between-group comparison analysis of players' ability to generate strength and power was conducted.

### 2.3 Measurements

#### 2.3.1 The screening protocol

The screening protocol was conducted by a licensed physiotherapist who evaluated the following factors with “YES” (1 point) and “NO” (0 points): (i) deviant passive and active range of motion in the knee joint, (ii) pain in the knee during squatting, lunging, or jumping, (iii) limping while walking, (iv) positive patellar The Royal London Hospital Test, (v) palpable or visible the knee joint effusion, (vi) positive Lachmann, varus/valgus stress test, patellar apprehension, or McMurray's/Apley's test, and (vii) hypo- or atrophic quadriceps on only one side (side difference with limb symmetry index <90%). Players receiving one point were immediately excluded.

#### 2.3.2 Anthropometrics

Body height (BH) and body weight (BW) were measured in a locker room, with privacy ensured for each player. BH was measured using a stadiometer (Hyssna M, Sweden). BW was measured by an electronic scale (Garmin Index S2 smart scale, 010-02294-12, UK). Subsequently, body mass index was calculated by dividing body weight (in kilograms) by the square of body height (in meters).

#### 2.3.3 Strength testing

To measure isokinetic leg strength, players performed a split squat on a Smith-like robotic resistance machine (1,080 Quantum Synchro; 1,080 Motion AB, Sweden) at a constant speed of 1 m/s in the concentric (upward) phase. During the test, players positioned their rear foot at a height equivalent to four 20 kg weight plates (i.e., 20 cm). After being positioned in a split squat with the Olympic barbell across the trapezius muscles, players were instructed to descend under control to a self-selected depth before ascending with maximal intent. They performed three maximal repetitions on each leg, and the concentric peak force (N) was recorded for each, with the best result used for the analysis. Based on this, the relative peak force (N/kg) was calculated for each player and used in the analysis. Given that players had previously performed the test, no familiarization was performed, only a standardized warm-up consisting of 8–10 min of Zone 2 cycling with a harder final minute to break a sweat, followed by hip mobility (90/90) and lumbar “leg roll”. Players then performed loaded split squats: 5 reps at a fixed weight, then 3–4 progressive weight increases before their max attempt. Emphasis was on a purposeful warm-up without wasting unnecessary energy.

#### 2.3.4 Power testing

A six-second “all-out” peak power test on the Wattbike Pro, (“Wattbike Peak 6 test” (Wattbike Ltd, Nottingham, UK)) was used to measure the players' peak power output (31). First, the saddle and handlebar height, and the distance between them were adjusted to achieve approximately full extension of the playeŕs knee when the foot is in the downward position and of the elbows when holding the handlebar. The feet were secured to the pedals by the clips. Players performed a 10 min standardized warm-up that consisted of a 6 min steady-state cycling at 50% of maximum capacity, followed by three 6 s acceleration phases at 70%, 80%, and 90% of maximum capacity performed at the beginning of each minute. Following 15 min, players continued pedaling at low intensity until full recovery was achieved. Air braking and magnetic resistance were then set to levels ten and one, respectively. The test then commenced, with players initiating pedaling with their dominant leg following a five-second countdown. During the test, players cycled at maximal intensity for exactly six seconds, with verbal encouragement provided throughout. The resistance is equivalent to 1,045 W at 130 rpm and increases by around 90–100 W for each 5 rpm increase in cadence (31).

#### 2.3.5 Ultrasound examination of the patellar thickening

To examine PTT, signs of thickening and width, ultrasonography was performed with an ultrasound device (GE Logiq E R10 2021, GE Logiq Ltd, USA) as recommended by the European Society of Musculoskeletal Radiology (32). The examination was performed once on both legs in the morning before any training or strenuous physical activity. Players lay in a supine position with the tested knee flexed to about 30 degrees. To minimize patellar locking and maintain a consistent, comfortable position, a cushion was placed under the popliteal space.

Ultrasound gel was applied to the front of the knee, just below the patella, to improve contact between the transducer (GE L8-18i-D Hockey Stick Linear Probe, GE Logiq Ltd, USA) and the skin and to obtain a clear view of the tendon fibers. For the long-axis scan, the linear probe was placed longitudinally just below the patella at the tendon origin and moved distally toward the tibial tuberosity at the tendon insertion. To inspect the whole tendon structure, the probe was moved medially and laterally. The short-axis view was obtained by rotating the probe transversely (33). The following pre-examination ultrasound setup was used: amplification level (i.e., gainer) of 45, frequency of 13 MHz, and depth and focal point of 7.5 mm. After a visual inspection of the tendon's most spread area, an ultrasound image was obtained. Dynamic provocation of Hoffa's fat pad was performed when needed to compress fat between the tendon and femur helping identify the exact transition between the patellar tendon and the fat pad. To increase measurement reliability, two independent examiners, with formal certification in musculoskeletal ultrasound, reviewed the ultrasound images. If the estimates differed, the lower value was used in the analysis. The cutoff value of >5 mm of patellar tendon thickness was set to indicate PTT (19, 20, 34). Ultrasound scanning proved to be a reliable method for assessing tendon thickness (35).

### 2.4 Statistical analysis

Descriptive statistics [mean **±** standard deviation (SD)] were calculated for all variables. The Shapiro–Wilk test was used to assess the normal distribution of the observed measurements. One-way ANOVA, followed by a *post hoc* Bonferroni test, was used to identify differences among the healthy group and the groups with unilateral and bilateral PTT for the observed variables. To evaluate the effect sizes (ES) for differences among the groups, partial eta squared values (*η*²) were used as follows: small ES: >0.02; medium ES: >0.13; large ES: >0.26 (36, 37). A paired-samples t-test was used to identify differences in peak force and relative peak force output between the left and right legs, separately within each observed group. Based on a power of 0.90 (*α* = 0.05), approximately seven players per experimental group and seven controls were needed to detect a 20% difference between unilateral PTT, bilateral PTT, and controls, with 20% considered the minimal clinically relevant difference (38). To account for potential dropouts, the study aimed to recruit at least 25 players. The statistical significance for all tests was set at *p* < 0.05. Statistical analyses were performed using SPSS®30.0 (IBM SPSS Statistics, New York, USA) for Windows.

## 3 Results

The Shapiro–Wilk test results indicated that the data for all outcome measures were normally distributed. Descriptive statistics were calculated for all outcome variables, including one-way ANOVA statistics (Table 2).

**Table 2:** Descriptive statistics (mean ± SD) and ANOVA differences between the healthy group and the groups with unilateral and bilateral PTT in strength and power performance.

Variable	HG (*n* = 8)	UPTTG (*n* = 8)	BPTTG (*n* = 12)	*p*	η²
	Mean ± SD	Mean ± SD	Mean ± SD		
LL Peak Force (N)	1,715.0 ± 215.3	1,717.4 ± 217.2	1,657.4 ± 277.6	0.822	0.016
RL Peak Force (N)	1,701.8 ± 211.8	1,664.4 ± 183.5	1,664.9 ± 266.3	0.929	0.006
LL Relative Peak Force (N/kg)	19.9 ± 2.1	20.2 ± 2.1	18.34 ± 2.4	0.163	0.135
RL Relative Peak Force (N/kg)	19.8 ± 2.4	19.6 ± 1.6	18.5 ± 2.5	0.378	0.075
Power (Watt)	1,752.0 ± 240.7	1,630.3 ± 141.6	1,674.5 ± 187.3	0.452	0.062
Relative Power (Watt/kg)	20.3 ± 1.7	19.2 ± 1.5	18.6 ± 1.1*	0.047	0.217

HG, Healthy group; UPTTG, Unilateral patellar asymptomatic tendon thickening group; BPTTG, Bilateral patellar asymptomatic tendon thickening group; SD, standard deviation; RL, Right leg; LL, Left leg; p, one-way ANOVÁs probability of between-group differences with Bonferroni *post-hoc* pairwise comparison test; η², Eta-squared effect size of observed differences;.

* Significantly different from the healthy group at *p* < 0.05.

### 3.1 Differences between the groups

One-way ANOVA with Bonferroni *post hoc* analysis revealed that the healthy group differed from the group with bilateral PTT only in relative power output. The healthy group showed higher relative power output than the group with bilateral PTT [F(2, 25) = 3.45, *p* = .047, medium ES]. For the remaining variables, no significant differences were identified among the groups.

### 3.2 Differences between the left and right legs in peak force

A paired-samples *t*-test showed no differences between legs within any observed group for peak force (N) and relative peak force (N/kg). In the healthy group, peak force of the left leg (1,715.0 ± 215.3 N) and of the right leg (1,701.8 ± 211.8 N) were statistically similar (*t* = 0.58, *p* = 0.29, *d* = 0.20) as well as for relative peak force (19.9 ± 2.1 *vs.* 19.8 ± 2.4 N/kg, *t* = 0.52, *p* = 0.31, *d* = 0.69). In the unilateral PTT group, the peak force of the left leg (1,717.4 ± 217.2 N) was higher than that of the right leg (1,664.4 ± 183.5 N), but the difference was not statistically significant (*t* = 1.23, *p* = 0.12, *d* = 0.18). On the contrary, within the same group, the relative peak force of the left leg was lower but not statistically significant (20.2 ± 2.1 *vs.* 19.6 ± 1.6 N/kg, *t* = 1.29, *p* = 0.12, *d* = 0.40). Similarly, the peak force of the left leg (1,657.4 ± 277.5 N) and the right leg (1,664.9 ± 266.3 N) did not differ significantly (*t* = 0.38, *p* = 0.35, *d* = 0.10) in the bilateral PTT group. The same was observed for relative peak force (18.4 ± 2.4 *vs.* 18.5 ± 2.5 N/kg, *t* = 0.45, *p* = 0.13, *d* = 0.69).

## 4 Discussion

There are several important findings that should be acknowledged. First, no between-group differences were observed in absolute or relative peak force, indicating preserved maximal LE strength across all groups. Second, players with bilateral patellar tendon thickening demonstrated lower relative power output compared with healthy players, whereas no power deficits were detected in the unilateral group. Third, no differences were found between the left and right legs within any group for relative peak force, suggesting maintained interlimb symmetry in force production. This study advances current understanding by demonstrating that asymptomatic patellar tendon thickening does not uniformly impair performance in elite athletes, but may lead to subtle, task-specific deficits, particularly when structural changes are bilateral. These findings highlight the importance of distinguishing between unilateral and bilateral tendon involvement and suggest that early functional consequences of tendon pathology may be detectable primarily in high-velocity power tasks rather than maximal strength.

The primary finding of this study was that elite ice hockey players with bilateral asymptomatic patellar tendon thickening exhibited reduced relative power output, while maximal strength and interlimb symmetry remained unaffected. These findings suggest that bilateral structural tendon alterations may selectively impair high-velocity power production without compromising maximal force-generating capacity. Conversely, unilateral patellar tendon thickening did not result in detectable impairments in either strength or power, indicating that localized or compensatory adaptations may preserve performance in asymptomatic athletes. Tendinopathies are known to impair strength, often via pain, with knee extension strength in patellar tendinopathy reported 4%–40% lower than in healthy controls (17). Against this background, the absence of between-group differences in relative peak force and the lack of interlimb asymmetries in the present study are noteworthy. Previous research has primarily examined symptomatic or chronic cases, in which strength deficits and altered limb loading are more apparent (15, 23). In contrast, athletes in this cohort were asymptomatic and training at an elite level, which may explain preserved strength and symmetrical force despite structural tendon changes. Similar findings have been reported in other athletic populations, where imaging-detected tendon abnormalities did not correspond to reduced strength or clinical symptom (39, 40). In addition, strength assessment methods vary across studies, with single-joint, open-chain tests frequently being used (41). In this study, a multi-joint, closed-chain split squat was chosen for its substantial knee loading and clinical feasibility, though this may limit comparability with other research. Previous studies show that squat- and lunge-based exercises generate high knee loads at 60–90° flexion and strong quadriceps activation, directly stressing the patellar tendon (42, 43). Moreover, as the Wattbike Peak 6 test is a bilateral task, the contralateral limb may compensate for deficits in the affected limb, potentially masking unilateral impairments. The absence of left/right power distribution data limits our ability to distinguish true preservation of function from compensatory mechanisms. However, ice hockey requires high bilateral proficiency with repeated, alternating force production from both limbs, and impairments may therefore become more evident during sport-specific dynamic movements not fully captured by the present test. Still, using a stationary cycling test to assess power may not fully reflect ice hockey–specific power demands, as skating involves predominantly horizontal force production and unilateral push-off patterns. Future studies may benefit from incorporating on-ice sprinting or skating-specific performance tests to improve ecological validity.

The observed reduction in relative power in the bilateral patellar tendon thickening group may be explained by alterations in tendon mechanical properties. Increased tendon thickness, disrupted collagen organization, and reduced tendon stiffness can impair the efficient transmission of force during rapid, explosive movements (44, 45). Because power production depends not only on maximal force but also on the rate of force development (46) and elastic energy transfer (47), compromised tendon function may disproportionately affect power-based tasks while leaving maximal strength relatively intact (45). In contrast to our results, other studies report lower isokinetic quadriceps strength in professional basketball players with patellar tendinopathy despite preserved jump performance (41). Variations in power assessment methodology may partly explain these discrepancies. In brief, the differences in power measurements between bicycle-based and jump-based methods indicate that cycling sprints and jump tests are not interchangeable (48). Cycling tests measure sustained power and take longer to reach peak power, whereas jump tests assess short-duration explosive movements and achieve peak power almost immediately. In the present study, maximal power was evaluated using the Wattbike Peak 6 test rather than a jump-based assessment. This test is a validated measure of maximal power output with particular sensitivity to peak power (31). Additionally, its ease of administration, low injury risk, and suitability for repeated testing make it especially practical in ice hockey settings, where the availability of multiple units allows for parallel testing (49).

Finally, the absence of performance deficits in the unilateral group supports the concept of functional compensation, whereby athletes may redistribute load or adopt neuromuscular strategies to preserve performance. This implies that factors beyond tendon structure alone, such as altered elasticity, movement patterns, or neuromuscular control, may influence functional outcomes (40, 50). In contrast, bilateral tendon involvement likely restricts compensatory capacity, potentially explaining the reduced power output observed in the bilateral group. The distinction between unilateral and bilateral tendon pathology remains underexplored and constitutes an important contribution of the present study.

Some limitations in the present study need to be acknowledged. First, given the cross-sectional study design, the interpretation of the observed differences should be interpreted with caution, as the design does not allow for the determination of causality or directionality. Because both tendon status and performance outcomes were assessed at a single time point, it was unclear whether reduced strength and power were a cause or a consequence of the asymptomatic tendon thickening. Longitudinal or prospective study designs would be required to clarify whether changes in tendon health lead to subsequent reductions in strength and power, or vice versa. Second, only tendon width/thickness was assessed and reported normal values for patellar tendon thickness vary widely. In the general population, values range from 3 to 5 mm (51), with greater variation among elite athletes. Individuals who later develop tendinopathy often exhibit thicknesses of ≥5 mm (39), and chronic tendinopathy has been associated with values exceeding 5.9 mm (52). However, lower mean values have been reported in healthy elite athletes across sports (19, 35). Given this variability, a cutoff value of 5 mm was used to indicate tendon thickening and reduce the risk of misclassification. Third, in the present study, there was an established relationship between one of the authors and the players, which may entail a risk of observer bias, particularly related to expectancy effects. To reduce this source of error, the players were anonymized using a random numbering system, and an external test administrator was responsible for the entire testing procedure. Additionally, the sample size was calculated to provide 90% power (*α* = 0.05) to detect a 20% difference between groups. However, the observed difference between the healthy group and the bilateral PTT group was approximately 8%, well below this threshold. Although statistically significant (*p* = .047), the study was not powered to detect effects of this magnitude, placing the finding in a lower-sensitivity range and increasing the likelihood of imprecision given the small sample sizes. The corresponding *post hoc* power (≈70%–75%) reflects this limitation rather than confirming robustness. Therefore, the observed effect should be interpreted with caution, as it may be of limited clinical relevance and requires confirmation in larger studies. In addition, applying Bonferroni correction in a small cohort, while methodologically conservative, may have further reduced sensitivity by increasing the likelihood of Type II errors (false negatives). Consequently, potentially meaningful differences may have gone undetected. Lastly, although the results suggested preserved symmetry in force production, asymmetries in power output cannot be excluded, as they were not measured.

## 5 Conclusion

The findings indicate that asymptomatic bilateral patellar tendon thickening may reduce relative power output in elite ice hockey players, despite preserved maximal strength and limb symmetry, whereas unilateral changes do not appear to impair performance. These results suggest that structural tendon alterations, particularly when bilateral, can lead to subtle deficits in power that are not detected through strength testing alone, highlighting the value of monitoring power-based performance to identify early functional changes. Longitudinal studies are needed to determine whether reduced power precedes symptom onset or increases the risk of future patellar tendon pain.

## Data Availability

The data used in this study contains sensitive participant information (since it contains elite athletes), and participants did not provide consent for public data sharing. Additionally, the current approvals from the Swedish Ethical Review Authority (DNR 2024-05136-01 and 2025-01700-02) do not include open data dissemination. Requests to access the datasets should be directed to the corresponding author.
